# Making sense of sensation: a framework of interoceptive attribution and appraisal with clinical applications

**DOI:** 10.1098/rstb.2025.0161

**Published:** 2026-11-10

**Authors:** Hannah Savage, Anne K. Felsenheimer, Vojtěch Smekal, Sarah N. Garfinkel

**Affiliations:** ^1^ Institute of Cognitive Neuroscience, University College London, London, UK; ^2^ Max Planck Institute for Human Cognitive and Brain Sciences, Leipzig, Germany

**Keywords:** interoception, attribution, appraisal, anxiety

## Abstract

Interoception provides a link between bodily states and cognitive and affective processes across both unconscious and conscious domains and is fundamental for a coherent sense of self. While previous research linking interoception to mental health has predominantly focused on interoceptive accuracy, attention, self-beliefs and insight, we argue that the higher-order interpretation of consciously perceived bodily signals plays a fundamental and underexplored role in this relationship. We propose a framework delineating two higher-order processes—interoceptive attribution and appraisal—which constitute the interpretation of interoceptive sensations that reach conscious awareness. We outline key factors that modulate attention, attribution and appraisal, and evidence the relevance of this framework to mental health conditions. Given that these processes are accessible to cognition, they represent an important target for existing psychotherapeutic interventions. We integrate such interventions within our framework, highlighting their potential to enhance interoception-based therapeutic approaches.

This article is part of the discussion meeting issue ‘From being to identity: analysis and synthesis of the self’.

## 1. Introduction

The origins of subjective experience are thought to be a complex interplay between the sensory information the brain receives—about the body and the external environment—and existing expectations and prior beliefs [1–4]. The inputs are shaped by context and interpretation and are integrated into mental states. Although much of this processing happens outside of conscious awareness, certain sensations can become accessible when attention is directed towards them, allowing for cognitive control and influence. Research across neuroscience and psychology shows that the ability to accurately interpret and evaluate selectively attended sensory information, including internal bodily sensations, is essential for healthy functioning and psychological wellbeing [5].

Interoception, formally defined as the sensing, interpretation and integration of signals originating from within the body [5], links bodily states and cognitive and affective processes. In contrast to the rapidly changing inputs from exteroceptive senses, such visceral signals are continuously present throughout life [6]. Interoception thereby provides a moment-by-moment mapping of the internal physiological landscape [5,7]. Converging evidence suggests that this ongoing interoceptive inference contributes to the unity of a felt self and to its subjective stability over time [6,8]. In turn, this implicit, bodily self may form a scaffold for the emergence of higher-order, conscious self-concepts that incorporate attitudes, beliefs and identity [9].

Interoception has been delineated into numerous dimensions ([10]; see figure 1) that range from unconscious levels, such as (i) the neural representation of these signals within the central nervous system, (ii) the strength of the afferent signals in the periphery, and (iii) the preconscious impact on the processing of external stimuli, to conscious levels. The latter include (iv) interoceptive accuracy, defined as correct and precise monitoring, such as the correspondence between objectively measured physiological events and individuals’ reported experience of those events, (v) the respective self-reports or interoceptive beliefs regarding those perceived signals (independent of how accurately these signals are sensed), and (vi) the correspondence of the accuracy and beliefs, referred to as interoceptive insight. Lastly, higher-order dimensions include (vii) interoceptive attention, as both a habitual tendency to attend to bodily signals and the ability to do so when instructed, and (viii) the interpretation of the source and nature of these sensations. In contrast to other interoceptive domains, and despite historic and recent findings evidencing its importance [12–14], relatively little attention has been paid to this final facet of interoceptive processing. Herein, we propose a framework in which the interpretation of the source and perceived nature of consciously experienced sensations can be delineated into two distinct processes: attribution—determining what caused the sensations and appraisal—determining the meaning of the sensations to the self. We argue that these processes are of central importance to understanding how interoception relates to subjective experiences, especially for clinically relevant symptoms such as anxiety, and that they may act as the mechanism of action for some existing interoception-related therapies.

**Figure 1.**
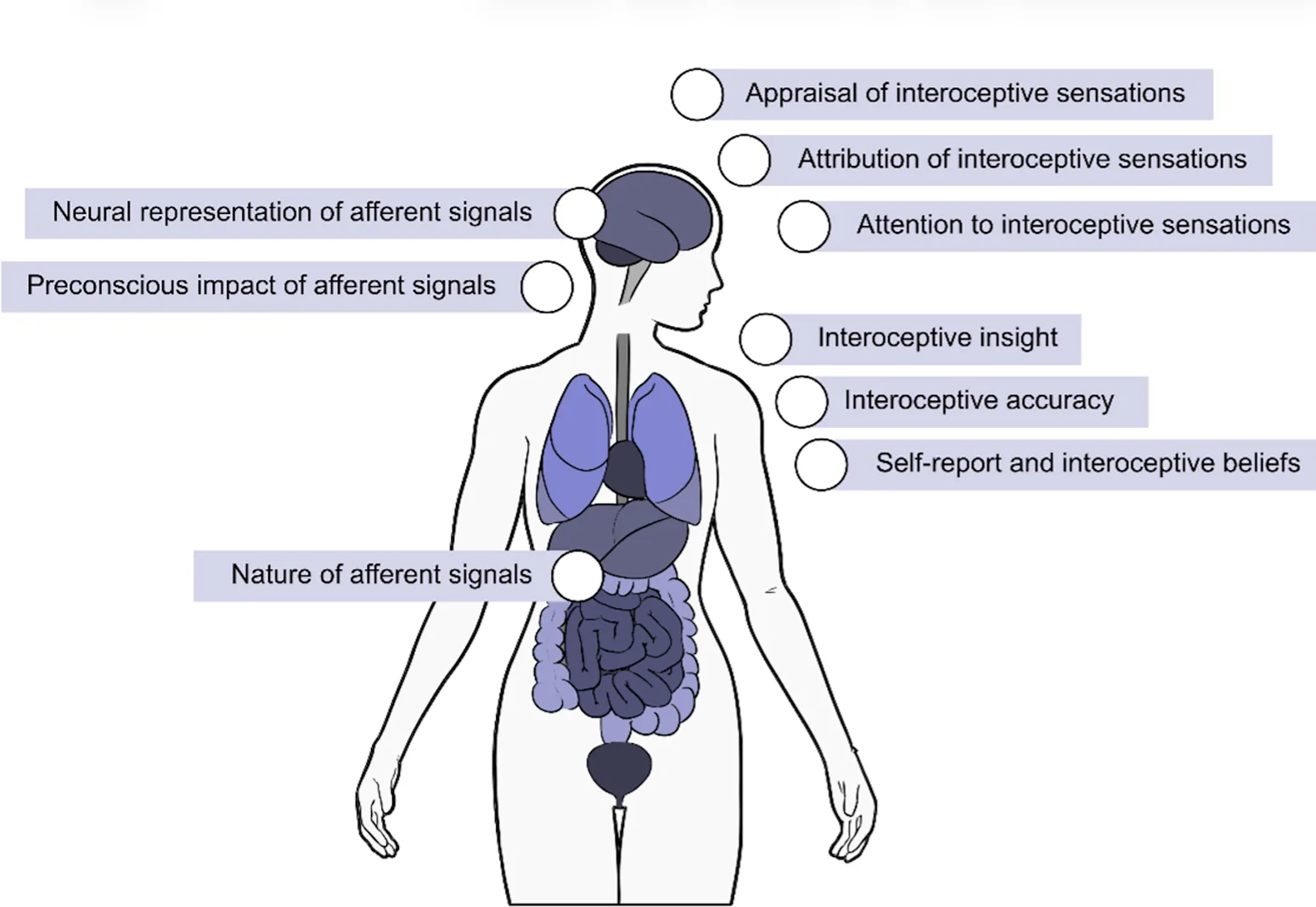
Updated representation of dimensions of interoception. Figure presenting the dimensions of interoception with the two processes focused on in this article, appraisal and attribution, in the top right of the figure. We do not suggest that appraisal and attribution happen exclusively during conscious interoceptive processing; however, that is the focus of the present paper. Figure adapted from Suksasilp & Garfinkel [10] and Greenwood & Garfinkel [11].

## 2. Attending to a sensation

Unless we deliberately direct attention to it, most of the time we do not consciously attend to an interoceptive signal. We typically operate without explicit awareness of the sensations coming from inside our body, prioritizing exteroceptive environmental demands. For instance, constant heartbeat monitoring while reading a text would be highly disruptive. However, interoceptive signals can spontaneously ‘breakthrough’ to awareness without prior attentional deployment. This typically occurs when something unexpected happens (e.g., sudden palpitations), even if the expectation was an implicit one. Such instances can be explained by predictive coding accounts of interoception.

According to predictive coding accounts, the brain generates probabilistic predictions about the internal bodily state and compares these with incoming interoceptive input. The difference between the prediction and the input is referred to as a prediction error, which is used to update and refine internal estimates of bodily states and adjust them accordingly [2–4]. Homeostatic processes can be regulated by these prediction errors, and pre-emptive predictions enable the brain to perform allostasis, optimizing energy efficiency by anticipating and meeting ongoing metabolic and survival needs [7]. Usually, this process occurs unconsciously, but when prediction errors are high, conscious attention may be drawn to the incoming internal and external sensory information in an effort to better understand what is happening and why our predictions were wrong. Additionally, explicit and verbalized priors or expectations (‘I am sick; my temperature should rise’) can likewise prompt input gathering on a conscious level (checking body temperature). Importantly, implicit signal management at ‘lower’ levels and explicit interoceptive processes at ‘higher’ levels are not isolated loops, but circularly interact [15]. For example, explicit attention can upregulate implicit interoceptive precision, while large predictive errors amplify metacognitive access.

Regardless of how we become aware of an interoceptive signal—through deliberate attention or an implicit mismatch with prior expectations—we propose that once we attend to it, two key questions emerge: why did the signal occur? and what does it mean for me (e.g. is it good, neutral or bad)?

## 3. Conscious and subconscious processing of sensations

Implicit interoceptive processes shape our emotions and perceptions continuously, even when we do not consciously pay attention to them. For instance, both tactile perception and active movement are influenced by the concurrent status of the heart in its constant dilation and contraction, with tactile and painful stimuli being attenuated [16] and movement initiation increased during systole [17]. Thus, without us consciously perceiving our heartbeat, it still shapes our cognition and perception. Even higher-level mental experiences such as fear processing [18], social pain and racial stereotypes [19] are shaped by the cardiac cycle. This extends to other bodily signals such as respiration, which affects cognition and fear discrimination [20,21]. Hence, interoceptive signals (e.g. the cardiac cycle) can shape pre-reflective subjective experiences (e.g. fear) without categorizing the signals themselves.

This paper focuses on the cognitive processing once we explicitly pay attention and perceive, or believe to perceive, a bodily signal. In contrast to the preconscious influence of bodily signals on our perception and emotions, it focuses on explicit moments of feeling that one can verbalize. Importantly, this also applies to signals that we think are not there when we assume they should be, e.g. noticing the *absence* of an increased heart rate when starting to exercise. In the case of our framework, whether the perceived signal is real (i.e. matches the true signal well) is irrelevant for the engagement of ‘higher-order’ attribution and appraisal processes on a conscious level. Despite the presence of a signal increasing detection likelihood, whether a perceived signal matches bodily reality is irrelevant for triggering attribution and appraisal. Conversely, both processes in turn have downstream consequences for bodily signals—appraisal and attribution can amplify or suppress existing signals and generate new ones (e.g. anxiety-caused tachycardia after an assumed ‘skipped heartbeat’).

## 4. The higher-order structure of interoceptive attribution and appraisal

Once consciously attended to, interoceptive signals, whether accurately or inaccurately perceived, can be attributed to a cause and appraised. This is done relative to an individual's physical and emotional psychosocial goals to inform the labelling of current and future emotional states, behaviour change and decision-making [22]. We propose that this is divided into two processes: attribution and appraisal (figure 1). We define attribution of interoceptive signals as determining the cause of interoceptive sensations based on an individual's prior and current internal and external context—why did this sensation happen? Appraisal is the process of determining the valence of the interoceptive signals—is this good, neutral or bad for me?

### (a) Attribution

The importance of attributing cause or relational meaning to consciously perceived interoceptive signals is not new; it is a central proposition of Schachter & Singer's two-factor theory of emotion [14], Zillmann's ‘excitation transfer’ theory [23], as well as attribution theories [24,25], and other emotion theories, including the theory of constructed emotion [26] or the theory of constructed mind approach [1], more generally. In a series of ‘misattribution of arousal’ studies, Schachter & Singer, and others, induced hedonically neutral autonomic arousal using injections (adrenaline versus placebo) or bridges (high, wobbly suspension bridge versus a low, wide concrete bridge), showing that perception of heightened physiological arousal was not sufficient to elicit specific emotional states. Rather, subjectively reported emotions required an additional cognitive process (so-called ‘cognitions’; [14]) in which the cause of the perceived physiological response was established based on available external contextual cues [27,28]. When contexts are novel, instructions alone can be used to induce misattribution of physiological symptoms to non-emotional sources, and while the influence of instructions is weaker in a familiar context, *a priori* expectations or explicit instructions can still have an effect. For example, expecting heightened physiological arousal has been shown to decrease the intensity of subsequently evoked emotional states [14]. Interestingly, the misattribution effect is probably attenuated in highly emotive or physiologically threatening situations [27], when attribution probably works subconsciously without significant higher-order influence. We argue that ‘propensity to use’ interoceptive signals, an interoceptive domain proposed by Murphy [29], fits within our attribution of interoceptive signals process. We agree that there may be large individual differences, as Murphy suggests, in the relative use of internal versus external cues for attributing cause, which probably fluctuates at both the state and trait level. When interoceptive sensations are perceived to occur with no clear single cause, it is hypothesized that an ‘expanded search’ may result in the physiological change being attributed to multiple potential causes [30,31]. The weighting of these potential causes may be related to prior experiences or motivational biases, and the resulting uncertainty probably has significant consequences for the appraisal [32].

### (b) Appraisal

An interoceptive sensation is appraised in relation to the psychosocial goals of the individual at that moment in time. This process takes the sensation and potentially its attribution, and ascribes meaning with respect to the self. Cognitive biases and other situational factors, referred to as ‘appraisals’ in appraisal theory—such as certainty, goal conduciveness and perceived controllability—play a role in shaping the appraisal [33]. This appraisal is integrated into larger self-schema [34], and used to situationally categorize experiences as emotions (see the theory of constructed emotion [1]) with implications for decision-making and behaviour [11]. When focal attribution is not successful and, as a result, multiple potential attributions are put forward to be appraised, interoceptive sensations are more likely to be appraised negatively [31]. The presence of multiple possible attributions increases uncertainty about the source of the sensation, which may be particularly aversive for individuals high in intolerance of uncertainty. In such cases, ambiguity itself may bias appraisal towards more negative interpretations. While this biased appraisal may optimize the likelihood of survival (i.e. better safe than sorry), it probably has unintended consequences, such as contributing to the manifestation and maintenance of mental health symptoms. Conversely, a positive appraisal of an objectively negative cause can occur as well, for instance, when individuals reinterpret a stressful or aversive bodily state as meaningful, motivational or indicative of growth—demonstrating the flexibility and context-dependence of interoceptive appraisal processes.

## 5. Predictions resulting from the framework

We postulate attribution and appraisal to be distinct yet interacting processes. Attribution represents an epistemic process concerned with accuracy and the reduction of uncertainty, whereas appraisal can be understood as a pragmatic process primarily concerned with value and goal-directed action. Thus, while attribution concerns the ‘why’ of a bodily signal, appraisal determines ‘what it means for me’, embedding it within a valence-associated evaluation that shapes the resulting subjective experience.

If these two are indeed separable processes, then several predictions follow from that assumption. Firstly, it should be possible to observe clinical or experimental cases in which one process is impaired while the other remains intact. For example, an individual might show attributional impairment with a functional appraisal: they may be unable to identify the cause of their racing heart (high attributional ambiguity) but still maintain a neutral or positive appraisal if contextual cues point to such an appraisal. Conversely, an intact attribution could be coupled with dysfunctional appraisal. This may be observed in individuals with anorexia nervosa, who correctly identify a hunger signal (accurate attribution) but appraise it as positive or desirable, given that it aligns with the goal of maintaining low body weight.

Secondly, there should be cases where the same attribution leads to different appraisals, as well as vice versa. For instance, when experiencing a racing heart while running in the park, one might attribute the cause to physical exertion and subsequently appraise it as a normal and healthy response to exercise. Yet, the same attribution (‘I am running in the park’) could lead to a different appraisal (‘I am unfit’), depending on the individual's goals and self-evaluative context.

Finally, we assume a cyclical feedback loop between implicit and explicit processes. Both attribution and appraisal can influence subsequent perceptual and bodily processes. An initial appraisal can shape attentional focus on interoceptive cues, generating new sensory evidence that prompts the individual to revise the prior attribution. For instance, an initial sensation of chest tightness might be attributed to stress; if the corresponding appraisal is one of high threat, the resulting anxiety may amplify physiological arousal, leading the individual to reattribute the symptom to a cardiac cause. Similarly, different attributions can have differing effects. If a burning eyes sensation is attributed to a lack of sleep, this will bias subsequent appraisal and cognition differently than if it is attributed to irritant particles from a nearby construction site. From this dynamic, we predict that altering appraisal and attribution should have measurable consequences on subsequent bodily sensations and interpretations (box 1).

**Box 1. Neural circuitry**

Our higher-order framework of interoceptive attribution and appraisal complements the Embodied Predictive Coding [2] model of brain function as well as a proposal by Koban *et al.* [34]. They hypothesize that the interoceptive-salience network facilitates reactive detection and contextualization of novel events or information when adjusting states may be required and that (unconscious or conscious) default mode and, specifically, ventromedial prefrontal cortex-mediated appraisal processes continuously regulate autonomic function and behaviour.

**(a) Attribution**

We propose that the attribution of interoceptive sensations is strongly related to the processing and contextualization of the salience and/or arousal of the perceived interoceptive sensations. Brain regions that show an increased BOLD signal when participants are instructed to attend to interoceptive signals are also involved in the salience network and in processing prediction errors [35]. More specifically, via the brainstem, incoming viscerosensory input signals converge in subcortical structures, such as the ventromedial nucleus of the thalamus and the nucleus of the solitary tract [36]. In turn, the neurons in these structures project to higher brain regions, including the posterior insular cortex where dimensionality reduction is thought to occur along the granular gradient to the anterior insular cortex [2,37,38], the anterior cingulate cortex and the somatosensory cortex, where these signals are integrated with contextual information to attribute cause to the interoceptive signal.

**(b) Appraisal**

We hypothesize that the regions which support the appraisal process are mostly those which perform self-related and valence judgments. This probably includes brain regions within the default mode network such as the ventromedial prefrontal cortex—thought to be central in computing subjective value by integrating knowledge structures and latent states represented in diffuse cortical areas [34,39,40]—as well as the neighbouring medial and lateral prefrontal cortex, anterior and posterior cingulate cortex, and precuneus, as well as the temporoparietal junction and cerebellum [34,35]. As detailed in the self-in-context model, these regions are well positioned to enact mobilization or regulation of autonomic and endocrine systems which, in collaboration with their influence on decision-making, guide the future state of the self [34].

## 6. Factors that modify attribution and appraisal

There are likely to be numerous factors, states and traits that influence the input to and outcome of attribution and appraisal processes. Below, we detail how altered motivations, high levels of anxiety sensitivity, and an intolerance of uncertainty may play such a role.

### (a) Intrinsic and extrinsic drive

The prior expectations you have for any given sensation, be that internally or externally driven, are likely to shape the attribution ascribed to it. Both involve a voluntary attention to the body, but they differ in what triggers this attention: the source of this drive either stems from an intrinsic cause (i.e. self-generated motivation) or an extrinsic cause (e.g. context-activated motivation).

An intrinsic driver to pay attention to your heartbeat may be to determine your level of exertion, thereby attributing any change in cardiac sensation to the physical activity you are doing [41]. Alternatively, if you have been recently told by a doctor that you are at significant risk of a heart attack, you may be paying attention to your heartbeat in order to assess your heart's functioning (an intrinsic motivation) and could attribute any sudden change in cardiac sensation to a heart attack. This notion draws upon existing research, such as aspects of work examining bodily threat monitoring in the context of a ‘fear of cancer return’ [42]—a phenomenon in which people living with and/or beyond cancer have a motivation to detect something being wrong with the body (i.e. as indicating cancer recurrence) and so persistently pay attention and (mis)attribute interoceptive sensations to a cancer-related cause.

External influences may also shape your attention to interoceptive sensations. The role of extrinsic motivators is relevant to clinical conditions that primarily occur in certain situations or under certain conditions, such as performance anxiety, social anxiety and specific phobias, which are driven by task requirements, social contexts and the presence of phobic stimuli in the environment, respectively. In addition, von Mohr *et al.* [43] show that, when biased, these external pressures may systematically alter interoceptive attribution and appraisal: in their study, interoceptive accuracy was linked to body satisfaction exclusively during late puberty, aligning with the heightened perceived social pressure to be thin [44].

Both intrinsic and extrinsic motivation involve self-relevance, but they differ in what is being inferred (internal versus external threat) and how the state is activated (trait-like versus situation-bound). To position this drive construct within a testable computational Bayesian framework, drivers could be modelled as strong and specific attribution priors—e.g. noticing only stomach cramps as a symptom of stomach cancer return—or flatter and more non-specific priors—e.g. noticing a range of bodily sensations as a sign of stomach cancer return, such as tiredness, nausea or indigestion. The specificity and flexibility of these priors may be clinically relevant and a target for future interventions.

### (b) Anxiety sensitivity

A negative bias to interpret interoceptive signals, especially those associated with anxiety, as aversive or life-threatening—known as anxiety sensitivity [45–47]—is a key feature of anxiety and anxiety-related disorders such as panic disorder [48–51]. These biased beliefs modulate the appraisal individuals make of their otherwise neutral physiological signals. Studies show that people with high levels of anxiety sensitivity rate their subjective anxiety level higher than people with low levels of anxiety sensitivity, despite no objective differences in physiological arousal [52–55]. For example, using a threat conditioning paradigm with CO_2_-enriched air as the unconditioned stimulus, Forsyth *et al.* [52] found comparable levels of conditioned and unconditioned responding in autonomic domains (skin conductance and heart rate) across participants with low-, moderate- and high-levels of anxiety sensitivity. By contrast, subjective reports of distress and overall panic symptoms clearly distinguished the high-anxiety sensitivity group from the others. Interestingly, this study also reported sex effects in distress ratings, whereby females reported higher levels of distress than males, which may suggest that this construct may play a role in higher rates of anxiety disorders in women [52]. While this study did not specifically disentangle attribution from appraisal, in this experimental context participants learned to attribute the sensations to the presence of the unconditioned stimulus by its association with the conditioned stimulus (as indexed by their successful discrimination learning), and the appraisal for subjective experience construction is what differed between groups. High levels of anxiety sensitivity probably also interact with motivational biases to shape attribution priors—once an individual is afraid of bodily signals, they are probably motivated to attend to forthcoming signals (i.e. threat monitoring) such that the negative emotional state is easily evoked and further maintained or exacerbated.

### (c) Intolerance of uncertainty

People can differ from each other in whether they attribute a sensation to a single cause (focal attribution) or whether they entertain many different options. While focal attribution provides a clearer course of action for subsequent cognition, flexibly switching between ranges of potential attributions can be adaptive, particularly in the event of noisy and uncertain contextual information.

The preference for focal or multiple attributions is most likely shaped by intolerance of uncertainty. This transdiagnostic factor, thought to contribute to the genesis and maintenance of many anxiety disorders [56–59], refers to ‘an individual's dispositional incapacity to endure the adverse response triggered by the perceived absence of salient, key or sufficient information, and sustained by the associated perception of uncertainty’ [59, p. 335, 60]. People who experience intolerance of uncertainty may feel distress when thinking about myriad or unknown causes of interoceptive sensations. This, in turn, modulates both appraisal and attribution: we hypothesize that individuals who experience a high intolerance of uncertainty are more likely to be bothered or upset by the seemingly unknown cause of the interoceptive sensations and thus negatively appraise them, irrespective of the probability or true likelihood of negative outcomes [30,31]. In addition, they may be more biased to tend to focal attributions to relieve distress.

## 7. Clinical relevance of interoceptive attribution and appraisal processes

We posit that individual differences in attribution and appraisal processes are of clinical relevance, not only contributing to the aetiology and maintenance of mental health conditions, but also as the target of existing interventions. Numerous studies that elicit emotional reactions, such as those that use fear conditioning paradigms or which show emotional faces or videos, report no or few differences in physiological responding between individuals with an anxiety-related disorder compared to unaffected controls [61–64]. By comparison, group differences in subjective reports are more frequently reported, which suggests that it is higher-order interoceptive domains wherein differences between patients and controls emerge [61–64]. More specifically, we propose that alterations in the attribution and/or appraisal of interoceptive signals, and their interaction with motivation, anxiety sensitivity and intolerance of uncertainty could potentially explain symptomatology related to anxiety, anhedonia and other mental health symptoms.

### (a) Panic disorder

Panic disorder is an anxiety disorder characterized by recurrent, unexpected panic attacks; a panic attack is a paroxysmal surge in heightened (perceived) physiological arousal (e.g. palpitations, pounding heart, accelerated heart rate, sweating, trembling, shaking, sensations of shortness of breath or smothering, feeling of choking, chest pain or discomfort, nausea or abdominal distress) and thought (e.g. derealization or depersonalisation, fear of losing control or ‘going crazy’, fear of dying; [65]). Thus, the two cornerstones of this diagnosis are the sudden and unexpected occurrence of physiological arousal and the excessive and disabling fear of the bodily sensations of arousal.

People with panic disorder often misattribute the interoceptive sensations they experience to a life-threatening cause. Then, believing themselves to be at significant risk of death, they form a negative appraisal of these symptoms. This, in turn, motivates increased attending to and noticing of future physiological changes, and perpetuates a cycle of heightened arousal and negative affect. Panic disorder symptom severity is highly correlated with anxiety sensitivity [66] independent of trait anxiety [47], and prospective studies with healthy adults [47,67] and adolescents [68] indicate that anxiety sensitivity predicts the future occurrence of anxiety symptoms and panic attacks [47]. While this may suggest that the appraisal of interoceptive sensations is disrupted, had interoceptive sensations not been misattributed in the first place, they would probably not be subsequently feared.

### (b) Generalized anxiety disorder

The intolerance of uncertainty model of generalized anxiety disorder emphasizes threatening interpretations of ambiguous or uncertain information as a pillar in the aetiology of this condition [32]. We hypothesize that instances of uncertain attribution of interoceptive sensations may facilitate negative appraisals via their interaction with an individual's dispositional trait of being intolerant to uncertainty. Intolerance of uncertainty is highly correlated with worry, a core symptom of generalized anxiety disorder, as well as overall generalized anxiety disorder symptom severity [69,70].

An alternative or complementary hypothesis pertains to ‘negative alliesthesia’, whereby individuals with generalized anxiety disorder experience repeated exposure to their internal milieu in stressful situations, which increases the negative valence of the internal milieu more generally. This may result in an inability to differentiate between constantly fluctuating interoceptive afferents and those associated with aversive stimuli, an example of disrupted attribution [71]. This is additionally supported by literature on interoceptive conditioning, wherein low-level somatic sensations of arousal or anxiety become associated with higher levels of subjective anxiety, such that the occurrence of normal physiological arousal is appraised negatively and thus engenders an anxious state [72]. If this process is occurring continuously in response to ongoing homeostatic fluctuations, it is conceivable that this would contribute to the sense of general unease and discomfort experienced by people with generalized anxiety disorder.

### (c) Social anxiety disorder

Social anxiety is an anxiety disorder characterized by a fear of negative evaluation by others, whereby people with social anxiety avoid social situations or endure them with significant discomfort. A key concern of people with social anxiety is the autonomic arousal they are experiencing in social settings, and their concern that others will notice their anxiety symptoms and humiliate, judge or reject them as a result [65]. People who experience high levels of social anxiety symptoms pay more attention to physiological information during anxiety-inducing social situations than people with lower levels of social anxiety, despite there often being minimal objective differences [73–75]. Arnold *et al.* [76] propose, based on work by Werner and colleagues [32,77], that individuals with lower interoceptive accuracy may be more likely to attribute physiological responses evoked by stressful social situations to intrinsic traits or flaws, as opposed to the external social situation. The increased and excessive self-focus experienced by people with social anxiety in social situations (i.e. both extrinsic motivation to attend to the signals and rumination on their intrinsic attribution) can be cognitively demanding and detract from individuals’ abilities to engage optimally in social interactions [75,78,79]. Furthermore, the appraisal of the interoceptive sensations gets entangled with biased theory-of-mind processes (‘can they tell I am anxious?’) that may ultimately make the sensations something to be feared.

### (d) Anhedonia and depression

Anhedonia is an experience of ‘diminished interest or pleasure in all, or almost all, activities most of the day, nearly every day (as indicated by either subjective account or observation)’ [65, p. 202]. It constitutes a hallmark symptom of depression, but it is also common in many other disorders. Given its central role in altered affective experience, researchers have examined whether anhedonia and related depressive symptoms are associated with changes in interoceptive processing. Yet, results on the detection accuracy of heartbeats in individuals with depressive symptoms are often mixed in experimental set-ups [80–82]. One possible reason for this inconsistency lies in the experimental context: in laboratory tasks, participants are explicitly instructed to focus on their bodily signals (e.g. during heartbeat counting), while in everyday life such interoceptive attention is typically self-initiated and driven by motivation. From this perspective, anhedonia may not reflect a deficit in interoceptive ability *per se*, but rather a disrupted appraisal, leading to reduced motivation to attend to bodily signals. People with anhedonia show diminished pleasure, as well as impairments in reward anticipation and learning [83]. If an individual does not expect a situation to be rewarding, they may not ‘reach out’ to capture the internal physiological shifts that normally signal excitement or appetitive desire. Consequently, the individual misses the internal evidence required to construct a positive emotional state.

Recent research on eating behaviour in anhedonia supports this perspective [84]. Individuals with high anhedonic traits subjectively reported no general deficits in noticing bodily signals, but differed in how they evaluated them, reporting lower physical well-being after consuming healthy snacks. They also engaged in less intuitive eating, which relies on responding to internal cues of hunger [85]. This pattern points to a reduced motivation to attend to or act upon bodily signals. Even when interoceptive cues are accurately detected and correctly attributed (e.g. ‘my heart is racing because I am opening a gift’), anhedonia may disrupt their positive appraisal (‘it doesn't matter’). In non-anhedonic individuals, higher interoceptive accuracy tends to enhance emotional intensity [86]. By contrast, in anhedonia this link was reversed, with greater interoceptive accuracy coinciding with dampened subjective arousal [87]. Rather than appraising a racing heart as a sign of positive excitement, the anhedonic response could dampen the arousal by appraising it as meaningless.

## 8. Modifying attribution and appraisal in therapeutic interventions

If the attribution and appraisal of interoceptive signals are as central to certain mental health conditions as we propose, this has important clinical implications, as these processes are amenable to therapeutic interventions. Below, we briefly discuss some well-established interventions, which we map within our higher-order structure of interoceptive attribution and appraisal framework.

### (a) Interoceptive exposure therapy

Interoceptive exposure therapy aims to help a patient learn to appropriately gauge how their bodily sensations match their perceived danger [88]. This enables them to identify the maladaptive thoughts and beliefs that are automatically associated with these signals (i.e. their anxiety sensitivity) and (re)learn that these sensations should no longer be considered alarming in the absence of other explicitly threatening stimuli [57]. By inducing physiological responses—by jogging, spinning, hyperventilation or breathing through a straw, for example—individuals experience a physiological challenge and direct their attention to the heightened bodily sensations [88]. They are then encouraged to correctly contextualize (attribute) and challenge their automatic negative beliefs (appraisal). This therapy may be augmented with biofeedback (see below) to help improve the accurate perception of the signals [89]. Historically, this intervention has been used as part of cognitive behaviour therapy to treat panic disorder, but there is accumulating evidence of its usefulness in treating a variety of anxiety-related disorders including generalized anxiety disorder, social anxiety disorder and obsessive-compulsive disorder [88,90]. Interestingly, in a case study reported by Boswell *et al.* [90, p. 427], a participant with a primary diagnosis of generalized anxiety disorder experienced ‘a minor reduction in the intensity of physical sensations, yet a more substantial decrease in subjective distress’, as well as a greatly reduced anxiety sensitivity score as a result of interoceptive exposure therapy. This suggests that the mechanism of action of this intervention may not be learning to decrease attention to these signals by way of exposure and habituation alone, but rather, or at least in part, helping individuals to more appropriately attribute and appraise bodily sensations when they occur [90].

### (b) Biofeedback

Biofeedback provides individuals with visual, acoustic or tactile feedback on their interoceptive signals, which is often used as part of a training protocol to teach individuals strategies to constrain these signals within optimal ranges [91,92]. For example, heart rate or heart rate variability biofeedback training harnesses the influence of paced breathing (breathing at 0.1 Hz, approx. six breaths per minute; or using an individualized resonance frequency [93]) to bring heart rate and respiratory oscillations in phase and modulate parasympathetic and sympathetic nervous activity. Given that the evidence for biofeedback improving heart rate variability beyond the intervention is inconclusive [92], it may be that training decreases anxiety and panic symptoms by altering higher-order levels of interoception [89,93,94]. Increasing the perceived controllability of physiological sensations with biofeedback training may help to decrease the likelihood of immediate negative appraisals by developing an understanding that unwanted or highly arousing signals can be actively regulated, managed and, if necessary, attenuated to a subthreshold level of awareness [93]. If these strategies are enacted at times of psychophysiological distress, positive physiological changes would probably be attributed to the intervention (i.e. ‘my heart rate has decreased because I am doing slow breathing’), and appraised positively (‘I am calming down’). A recent review suggested that short training periods may be sufficient for significant effects, and as such, the practice of heart rate variability biofeedback-mediated breathing strategies may be easy to grasp and apply to daily life [93].

### (c) Reappraisal

Reappraisal is a cognitively driven emotion regulation strategy that encourages a person to think about a situation, sensation or thought in a way that alters the emotional response [95]. ‘Cognitive appraisals are powerful tools that help shift negative stress states to more positive ones’ [73, p. 51]. The framework we propose fits well with existing research on emotion regulation [95,96] and more specifically cognitive reappraisal techniques [73]. For example, take an individual who is experiencing and attending to a number of interoceptive sensations—a racing heart, tense and churning stomach, and shallow breathing—which they attribute to them being about to give a public talk. They may at first appraise this to mean ‘I am anxious’, a negative emotional state known to impair functioning and performance [97]. Using arousal reappraisal strategies [73], this individual could reappraise the sensations as ‘I am excited’, a more positively valenced emotional state that shares features of high arousal and anticipation with anxiety, but has been shown to enhance performance [98–100].

Alternatively, using more traditional intrinsic emotion regulation strategies (called ‘cognitive change’ in Gross's model; [95]) they might aim to decrease their arousal by reappraising the sensations in a more neutral way: ‘my racing heart isn't a sign of anxiety; it means my body is preparing for the speech’ [73,95,101].

### (d) Mindfulness

Mindfulness-based training—defined herein as training that promotes awareness of the present experience (including interoceptive signals) without any judgement [102], or without attachment or cognitive elaboration [103]—has been shown to decrease anxiety and panic symptoms [104–106]. In this literature, ‘awareness’ is the process of continually monitoring the inner and outer environment, a concept that aligns most closely with ‘attention’ in our framework. Lima-Araujo *et al.* [107] found that a brief 3-day mindfulness training did not improve cardiac interoceptive accuracy (as in [108]), but rather improved interoceptive self-beliefs, as operationalized using the multidimensional assessment of interoceptive awareness (MAIA; [109]) where changes were specific to the attention subscale. This, in turn, was thought to mediate the observed decrease in state anxiety. It may be possible that the effects of mindfulness are domain-specific—see [110] for some improvements in respiratory interoceptive accuracy using a breath-based meditation—or work by changing the nature of the afferent signals themselves [111]. Alternatively, we propose that some individuals may have a maladaptive motivational bias, which skews attention, leading to improbable attributions and negative appraisals. Therefore, the efficacy of this intervention may lie in training individuals to allow interoceptive signals to hold attention without attaching further interpretations or judgments to their experiences. Another possibility, suggested by Kim *et al.* [105], is that mindfulness-based cognitive therapy may decrease anxiety (specifically panic symptoms) by decreasing intolerance of uncertainty. They suggest that this occurs as individuals learn to tolerate the inevitable uncertainty of internal signals throughout training. Similarly, we propose that, akin to interoceptive exposure therapy for heightened autonomic activity, mindfulness may help trainees unlearn conditioned negative associations with autonomic activity at rest or in states of normal fluctuations (see negative alliesthesia in generalized anxiety disorder above), provided they can endure the initial discomfort without harm [112].

## 9. Concluding remarks

Our framework extends the interoception dimensions proposed by Suksasilp & Garfinkel [10]. Specifically, we take their final dimension—attribution of interoceptive sensations, which encompassed both the interpretation of the source and nature of these sensations—and separate this into two distinct processes. One, interoceptive attribution, pertains to determining what caused these sensations, and the other, interoceptive appraisal, ascribes the meaning of the sensations to the self. In doing so, we draw upon many well-established fields of literature that we do not claim to cover in their entirety; from interoception to predispositions of personality and cognitive biases, and clinical insights and interventions. Each field holds a wealth of history and expertise as well as unique strengths and limitations of measurement and analysis. Additionally, there are likely to be other domains and modulators that we have not included, such as those explored in the appraisal theory of emotion [33], and parallel subconscious processes which we encourage future researchers to explore further. Here, we illustrate the application of our framework to anxiety-related conditions, but there are clear applications to other mental health and functional conditions that require further consideration; major depressive disorder may be associated with decreased intrinsic motivation to attend to interoceptive sensations, alexithymia may be associated with increased exteroceptive motivations and biased interoceptive attributions (to somatic causes, rather than emotional causes), and irritable bowel syndrome may be associated with increased intrinsic motivations, misattributions and negatively biased interoceptive appraisals. With this framework, we hope to inspire future work, which directly probes the interrelation of interoceptive attribution, interoceptive appraisal and their modifying factors, to better understand mental health and related clinical conditions (box 2).

**Box 2. Outstanding questions**

— Does improving accuracy, aligning self-beliefs or refining insight, as is achieved by an interoceptive training intervention [113], influence the subsequent attribution-appraisal process?
— Assuming somewhat comparable attribution-appraisal processes occur without conscious awareness, how do they relate to the consciously accessible processes?
— To what degree does individual difference in the use of internal or external information to perform attribution link to appraisals, and how does this relate to health and wellbeing [29]?
— Can we develop a test of interoceptive attribution and/or interoceptive appraisal that could be used to select optimal treatments?

## Data Availability

This article has no additional data.

## Theme

One contribution of 17 to a discussion meeting issue ‘From being to identity: analysis and synthesis of the self’.

## References

[1] Barrett LF , Lida T. 2024Constructionist approaches to emotion in psychology and related fields. In Routledge handbook of emotion theory (ed. Scarantino A), p. 361. New York, NY: Routledge.

[2] Barrett LF , Simmons WK. 2015 Interoceptive predictions in the brain. Nat. Rev. Neurosci. 16, 419–429. (doi:10.1038/nrn3950) PMC4731102 26016744

[3] Clark A . 2013 Whatever next? Predictive brains, situated agents, and the future of cognitive science. Behav. Brain Sci. 36, 181–204. (doi:10.1017/s0140525x12000477) 23663408

[4] Friston K . 2010 The free-energy principle: a unified brain theory? Nat. Rev. Neurosci. 11, 127–138. (doi:10.1038/nrn2787) 20068583

[5] Khalsa SS *et al.* 2018 Interoception and mental health: a roadmap. Biol. Psychiatry Cogn. Neurosci. Neuroimaging 3, 501–513. (doi:10.1016/j.bpsc.2017.12.004) PMC6054486 29884281

[6] Tsakiris M . 2017 The multisensory basis of the self: from body to identity to others. Q. J. Exp. Psychol. 70, 597–609. (doi:10.1080/17470218.2016.1181768) PMC5214748 27100132

[7] Quigley KS , Kanoski S, Grill WM, Barrett LF, Tsakiris M. 2021 Functions of interoception: from energy regulation to experience of the self. Trends Neurosci. 44, 29–38. (doi:10.1016/j.tins.2020.09.008) PMC7780233 33378654

[8] Damasio A . 2010 Self comes to mind: constructing the conscious brain. New York, NY: Pantheon Books.

[9] Southgate V . 2026 How does the developing mind construct a self? Phil. Trans. R. Soc. B 381, 20250158. (doi:10.1098/rstb.2025.0158)

[10] Suksasilp C , Garfinkel SN. 2022 Towards a comprehensive assessment of interoception in a multi-dimensional framework. Biol. Psychol. 168, 1083262. (doi:10.1016/j.biopsycho.2022.108262) 35026353

[11] Greenwood BM , Garfinkel SN. 2025 Interoceptive mechanisms and emotional processing. Annu. Rev. Psychol. 76, 59–86. (doi:10.1146/annurev-psych-020924-125202) 39423429

[12] Clemente R , Murphy A, Murphy J. 2024 The relationship between self-reported interoception and anxiety: a systematic review and meta-analysis. Neurosci. Biobehav. Rev. 167, 105923. (doi:10.1016/j.neubiorev.2024.105923) 39427810

[13] Lazarus RS . 1975 A cognitively oriented psychologist looks at biofeedback. Am. Psychol. 30, 553–561. (doi:10.1037/h0076649) 1147365

[14] Schachter S , Singer J. 1962 Cognitive, social, and physiological determinants of emotional state. Psychol. Rev. 69, 379–399. (doi:10.1037/h0046234) 14497895

[15] Villringer A , Nikulin VV, Gaebler M. 2025 Brain–body states as a link between cardiovascular and mental health. Trends Neurosci. 48, 766–779. (doi:10.1016/j.tins.2025.08.004) 40975669

[16] Al E , Iliopoulos F, Forschack N, Nierhaus T, Grund M, Motyka P, Gaebler M, Nikulin VV, Villringer A. 2020 Heart–brain interactions shape somatosensory perception and evoked potentials. Proc. Natl. Acad. Sci. USA 117, 10575–10584. (doi:10.1073/pnas.1915629117) PMC7229654 32341167

[17] Kunzendorf S , Klotzsche F, Akbal M, Villringer A, Ohl S, Gaebler M. 2019 Active information sampling varies across the cardiac cycle. Psychophysiology 56, e13322. (doi:10.1111/psyp.13322) 30620083

[18] Garfinkel SN , Minati L, Gray MA, Seth AK, Dolan RJ, Critchley HD. 2014 Fear from the heart: sensitivity to fear stimuli depends on individual heartbeats. The Journal of neuroscience: the official journal of the Society for Neuroscience 34, 6573–6582. (doi:10.1523/JNEUROSCI.3507-13.2014) PMC4012313 24806682

[19] Azevedo R , Garfinkel S, Critchley H, Tsakiris M. 2017 Cardiac afferent activity modulates the expression of racial stereotypes. Nat. Commun. 8, 13854. (doi:10.1038/ncomms13854) PMC5247580 28094772

[20] Perl O , Ravia A, Rubinson M, Eisen A, Soroka T, Mor N, Secundo L, Sobel N. 2019 Human non-olfactory cognition phase-locked with inhalation. Nat. Hum. Behav. 3, 501–512. (doi:10.1038/s41562-019-0556-z) 31089297

[21] Zelano C , Jiang H, Zhou G, Arora N, Schuele S, Rosenow J, Gottfried JA. 2016 Nasal respiration entrains human limbic oscillations and modulates cognitive function. The Journal of neuroscience : the official journal of the Society for Neuroscience 36, 12448–12467. (doi:10.1523/JNEUROSCI.2586-16.2016) PMC5148230 27927961

[22] Dunn BD , Galton HC, Morgan R, Evans D, Oliver C, Meyer M, Cusack R, Lawrence AD, Dalgleish T. 2010 Listening to your heart: how interoception shapes emotion experience and intuitive decision making. Psychol. Sci. 21, 1835–1844. (doi:10.1177/0956797610389191) 21106893

[23] Zillmann D . 2008Excitation transfer theory. In The International encyclopedia of communication(ed. Donsbach W). Malden, MA: Wiley-Blackwell. (doi:10.1002/9781405186407.wbiece049)

[24] Abramson LY , Seligman ME, Teasdale JD. 1978 Learned helplessness in humans: critique and reformulation. J. Abnorm. Psychol. 87, 49–74. (doi:10.1037/0021-843X.87.1.49) 649856

[25] Fitch G . 1970 Effects of self-esteem, perceived performance, and choice on causal attributions. J. Pers. Soc. Psychol. 16, 311–315. (doi:10.1037/h0029847) 5479133

[26] Barrett LF . 2017 The theory of constructed emotion: an active inference account of interoception and categorization. Soc. Cognit. Affect. Neurosci. 12, 1–23. (doi:10.1093/scan/nsw154) PMC5390700 27798257

[27] Cotton JL . 1981 A review of research on Schachter's theory of emotion and the misattribution of arousal. Eur. J. Soc. Psychol. 11, 365–397. (doi:10.1002/ejsp.2420110403)

[28] Dutton DG , Aron AP. 1974 Some evidence for heightened sexual attraction under conditions of high anxiety. J. Pers. Soc. Psychol. 30, 510–517. (doi:10.1037/h0037031) 4455773

[29] Murphy J . 2022 Propensity to use interoceptive signals: an important individual difference. Biol. Psychol. 171, 108326. (doi:10.1016/j.biopsycho.2022.108326) 35367309

[30] Del Popolo Cristaldi F , Mento G, Sarlo M, Buodo G. 2021 Dealing with uncertainty: a high-density EEG investigation on how tolerance of uncertainty affects emotional predictions. PLoS ONE 16, e0254045. (doi:10.1371/journal.pone.0254045) PMC8248604 34197554

[31] Maslach C . 1979 Negative emotional biasing of unexplained arousal. J. Pers. Soc. Psychol. 37, 953–969. (doi:10.1037/0022-3514.37.6.953)

[32] Werner NS , Duschek S, Mattern M, Schandry R. 2009 Interoceptive sensitivity modulates anxiety during public speaking. J. Psychophysiol. 23, 85–94. (doi:10.1027/0269-8803.23.2.85)

[33] Ellsworth PC . 2013 Appraisal theory: old and new questions. Emot. Rev. 5, 125–131. (doi:10.1177/1754073912463617)

[34] Koban L , Gianaros PJ, Kober H, Wager TD. 2021 The self in context: brain systems linking mental and physical health. Nat. Rev. Neurosci. 22, 309–322. (doi:10.1038/s41583-021-00446-8) PMC8447265 33790441

[35] Savage HS , Davey CG, Wager TD, Garfinkel SN, Moffat BA, Glarin RK, Harrison BJ. 2021 Neural mediators of subjective and autonomic responding during threat learning and regulation. Neuroimage 245, 118643. (doi:10.1016/j.neuroimage.2021.118643) PMC9533324 34699966

[36] Chen WG *et al.* 2021 The emerging science of interoception: sensing, integrating, interpreting, and regulating signals within the self. Trends Neurosci. 44, 3–16. (doi:10.1016/j.tins.2020.10.007) PMC7780231 33378655

[37] Craig AD . 2009 How do you feel – now? The anterior insula and human awareness. Nat. Rev. Neurosci. 10, 59–70. (doi:10.1038/nrn2555) 19096369

[38] Craig AD . 2011 Significance of the insula for the evolution of human awareness of feelings from the body. Ann. N.Y. Acad. Sci. 1225, 72–82. (doi:10.1111/j.1749-6632.2011.05990.x) 21534994

[39] Benoit RG , Szpunar KK, Schacter DL. 2014 Ventromedial prefrontal cortex supports affective future simulation by integrating distributed knowledge. Proc. Natl Acad. Sci. USA 111, 16550–16555. (doi:10.1073/pnas.1419274111) PMC4246308 25368170

[40] Sui J , Humphreys GW. 2015 The integrative self: how self-reference integrates perception and memory. Trends Cogn. Sci. 19, 719–728. (doi:10.1016/j.tics.2015.08.015) 26447060

[41] Wallman-Jones A , Perakakis P, Tsakiris M, Schmidt M. 2021 Physical activity and interoceptive processing: theoretical considerations for future research. Int. J. Psychophysiol. 166, 38–49. (doi:10.1016/j.ijpsycho.2021.05.002) 33965423

[42] Heathcote LC *et al.* 2023 The bodily threat monitoring scale: development and preliminary validation in adult and childhood cancer survivors. Psychooncology 32, 1885–1894. (doi:10.1002/pon.6236) PMC11311963 37916988

[43] Von Mohr M , Donaghy R, Ainley V, Moore I, Agyemang A, Tsakiris M. 2026 Tuning in: how interoception shapes body image across early adolescence. Phil. Trans. R. Soc. B 381, 20250159. (doi:10.1098/rstb.2025.0159)

[44] Stice E , Whitenton K. 2002 Risk factors for body dissatisfaction in adolescent girls: a longitudinal investigation. Dev. Psychol. 38, 669–678. (doi:10.1037//0012-1649.38.5.669) 12220046

[45] Reiss S . 1987 Theoretical perspectives on the fear of anxiety. Clin. Psychol. Rev. 7, 585–596. (doi:10.1016/0272-7358(87)90007-9)

[46] Reiss S , Peterson RA, Gursky DM, McNally RJ. 1986 Anxiety sensitivity, anxiety frequency and the prediction of fearfulness. Behav. Res. Ther. 24, 1–8. (doi:10.1016/0005-7967(86)90143-9) 3947307

[47] Schmidt NB , Lerew DR, Jackson RJ. 1997 The role of anxiety sensitivity in the pathogenesis of panic: prospective evaluation of spontaneous panic attacks during acute stress. J. Abnorm. Psychol. 106, 355–364. (doi:10.1037/0021-843X.106.3.355) 9241937

[48] Ehlers A , Breuer P. 1992 Increased cardiac awareness in panic disorder. J. Abnorm. Psychol. 101, 371–382. (doi:10.1037/0021-843x.101.3.371) 1500594

[49] Reiss S . 1991 Expectancy model of fear, anxiety, and panic. Clin. Psychol. Rev. 11, 141–153. (doi:10.1016/0272-7358(91)90092-9)

[50] Sandin B , Chorot P, McNally RJ. 2001 Anxiety sensitivity index: normative data and its differentiation from trait anxiety. Behav. Res. Ther. 39, 213–219. (doi:10.1016/S0005-7967(00)00009-7) 11153974

[51] Taylor S , Koch WJ, McNally RJ. 1992 How does anxiety sensitivity vary across the anxiety disorders? J. Anxiety Disord. 6, 249–259. (doi:10.1016/0887-6185(92)90037-8)

[52] Forsyth JP , Palav A, Duff K. 1999 The absence of relation between anxiety sensitivity and fear conditioning using 20% versus 13% CO_2_-enriched air as unconditioned stimuli. Behav. Res. Ther. 37, 143–153. (doi:10.1016/S0005-7967(98)00113-2) 9990745

[53] Shostak BA , Peterson RA. 1990 Effects of anxiety sensitivity on emotional response to a stress task. Behav. Res. Ther. 28, 513–521. (doi:10.1016/0005-7967(90)90138-9) 2076089

[54] Sturges LV , Goetsch VL. 1996 Psychophysiological reactivity and heartbeat awareness in anxiety sensitivity. J. Anxiety Disord. 10, 283–294. (doi:10.1016/0887-6185(96)00012-6)

[55] Sturges LV , Goetsch VL, Ridley J, Whittal M. 1998 Anxiety sensitivity and response to hyperventilation challenge: physiological arousal, interoceptive acuity, and subjective distress. J. Anxiety Disord. 12, 103–115. (doi:10.1016/S0887-6185(98)00004-8) 9560174

[56] Carleton RN . 2016 Fear of the unknown: one fear to rule them all? J. Anxiety Disord. 41, 5–21. (doi:10.1016/j.janxdis.2016.03.011) 27067453

[57] Grupe DW , Nitschke JB. 2013 Uncertainty and anticipation in anxiety: an integrated neurobiological and psychological perspective. Nat. Rev. Neurosci. 14, 488–501. (doi:10.1038/nrn3524) PMC4276319 23783199

[58] Whiting SE , Jenkins WS, May AC, Rudy BM, Davis III TE, Reuther ET. 2014 The role of intolerance of uncertainty in social anxiety subtypes. J. Clin. Psychol. 70, 260–272. (doi:10.1002/jclp.22024) 23843207

[59] Carleton RN , Thibodeau MA, Teale MJ, Welch PG, Abrams MP, Robinson T, Asmundson GJ. 2013 The center for epidemiologic studies depression scale: a review with a theoretical and empirical examination of item content and factor structure. PloS ONE 8, e58067. (doi:10.1371/journal.pone.0058067) PMC3585724 23469262

[60] Carleton RN . 2016 Into the unknown: a review and synthesis of contemporary models involving uncertainty. J. Anxiety Disord. 39, 30–43. (doi:10.1016/j.janxdis.2016.02.007) 26945765

[61] Anderson ER , Hope DA. 2009 The relationship between social phobia, objective and perceived physiological reactivity, and anxiety sensitivity in adolescent population. J. Anxiety Disord. 23, 18–26. (doi:10.1016/j.janxdis.2008.03.011) PMC2645715 18436426

[62] Grillon C , Pine DS, Lissek S, Rabin S, Bonne O, Vythilingam M. 2009 Increased anxiety during anticipation of unpredictable aversive stimuli in posttraumatic stress disorder but not in generalized anxiety disorder. Biol. Psychiatry 66, 47–53. (doi:10.1016/j.biopsych.2008.12.028) PMC2696581 19217076

[63] Yassa MA , Hazlett RL, Stark CEL, Hoehn-Saric R. 2012 Functional MRI of the amygdala and bed nucleus of the stria terminalis during conditions of uncertainty in generalized anxiety disorder. J. Psychiatr. Res. 46, 1045–1052. (doi:10.1016/j.jpsychires.2012.04.013) PMC3893050 22575329

[64] Yoris A *et al.* 2015 The roles of interoceptive sensitivity and metacognitive interoception in panic. Behav. Brain Funct. 11, 14. (doi:10.1186/s12993-015-0058-8) PMC4422149 25889157

[65] American Psychiatric Association . 2013 Diagnostic and statistical manual of mental disorders: DSM-5. Arlington, VA: American Psychiatric Association.

[66] Olatunji BO , Wolitzky-Taylor KB. 2009 Anxiety sensitivity and the anxiety disorders: a meta-analytic review and synthesis. Psychol. Bull. 135, 974–999. (doi:10.1037/a0017428) 19883144

[67] Schmidt NB , Lerew DR, Jackson RJ. 1999 Prospective evaluation of anxiety sensitivity in the pathogenesis of panic: replication and extension. J. Abnorm. Psychol. 108, 532–537. (doi:10.1037/0021-843X.108.3.532) 10466277

[68] Hayward C , Killen JD, Kraemer HC, Taylor CB. 2000 Predictors of panic attacks in adolescents. J. Am. Acad. Child Adolesc. Psychiatry 39, 207–214. (doi:10.1097/00004583-200002000-00021) 10673832

[69] Buhr K , Dugas MJ. 2006 Investigating the construct validity of intolerance of uncertainty and its unique relationship with worry. J. Anxiety Disord. 20, 222–236. (doi:10.1016/j.janxdis.2004.12.004) 16464706

[70] McEvoy PM , Hyett MP, Shihata S, Price JE, Strachan L. 2019 The impact of methodological and measurement factors on transdiagnostic associations with intolerance of uncertainty: a meta-analysis. Clin. Psychol. Rev. 73, 101778. (doi:10.1016/j.cpr.2019.101778) 31678816

[71] Paulus MP , Stein MB. 2010 Interoception in anxiety and depression. Brain Struct. Funct. 214, 451–463. (doi:10.1007/s00429-010-0258-9) PMC2886901 20490545

[72] Bouton ME , Mineka S, Barlow DH. 2001 A modern learning theory perspective on the etiology of panic disorder. Psychol. Rev. 108, 4–32. (doi:10.1037/0033-295X.108.1.4) 11212632

[73] Jamieson JP , Mendes WB, Nock MK. 2013 Improving acute stress responses: the power of reappraisal. Curr. Dir. Psychol. Sci. 22, 51–56. (doi:10.1177/0963721412461500)

[74] Mauss I , Wilhelm F, Gross J. 2004 Is there less to social anxiety than meets the eye? Emotion experience, expression, and bodily responding. Cogn. Emot. 18, 631–642. (doi:10.1080/02699930341000112)

[75] Rapee RM , Heimberg RG. 1997 A cognitive-behavioral model of anxiety in social phobia. Behav. Res. Ther. 35, 741–756. (doi:10.1016/S0005-7967(97)00022-3) 9256517

[76] Arnold AJ , Winkielman P, Dobkins K. 2019 Interoception and social connection. Front. Psychol. 10, 2589. (doi:10.3389/fpsyg.2019.02589) PMC6901918 31849741

[77] Werner NS , Kerschreiter R, Kindermann NK, Duschek S. 2013 Interoceptive awareness as a moderator of affective responses to social exclusion. J. Psychophysiol. 27, 39–50. (doi:10.1027/0269-8803/a000086)

[78] Mor N , Winquist J. 2002 Self-focused attention and negative affect: a meta-analysis. Psychol. Bull. 128, 638–662. (doi:10.1037/0033-2909.128.4.638) 12081086

[79] Mulkens S , de Jong PJ, Dobbelaar A, Bögels SM. 1999 Fear of blushing: fearful preoccupation irrespective of facial coloration. Behav. Res. Ther. 37, 1119–1128. (doi:10.1016/S0005-7967(98)00198-3) 10500324

[80] Dunn BD , Dalgleish T, Ogilvie AD, Lawrence AD. 2007 Heartbeat perception in depression. Behav. Res. Ther. 45, 1921–1930. (doi:10.1016/j.brat.2006.09.008) 17087914

[81] Eggart M , Lange A, Binser MJ, Queri S, Müller-Oerlinghausen B. 2019 Major depressive disorder is associated with impaired interoceptive accuracy: a systematic review. Brain Sci. 9, 131. (doi:10.3390/brainsci9060131) PMC6627769 31174264

[82] Pollatos O , Traut-Mattausch E, Schandry R. 2009 Differential effects of anxiety and depression on interoceptive accuracy. Depress. Anxiety 26, 167–173. (doi:10.1002/da.20504) 19152366

[83] Vrieze E , Pizzagalli DA, Demyttenaere K, Hompes T, Sienaert P, de Boer P, Schmidt M, Claes S. 2013 Reduced reward learning predicts outcome in major depressive disorder. Biol. Psychiatry 73, 639–645. (doi:10.1016/j.biopsych.2012.10.014) PMC3602158 23228328

[84] Frederiksen C , Victor Byrne D, Chan RCK, Vad Andersen B. 2025 The effect of anhedonic traits on snack food consumption and interoception of food-related pleasure and bodily sensations. Food Qual. Preference 122, 105282. (doi:10.1016/j.foodqual.2024.105282)

[85] Herbert BM , Blechert J, Hautzinger M, Matthias E, Herbert C. 2013 Intuitive eating is associated with interoceptive sensitivity. Effects on body mass index. Appetite 70, 22–30. (doi:10.1016/j.appet.2013.06.082) 23811348

[86] Wiens S , Mezzacappa ES, Katkin ES. 2000 Heartbeat detection and the experience of emotions. Cogn. Emot. 14, 417–427. (doi:10.1080/026999300378905)

[87] Dunn BD , Stefanovitch I, Evans D, Oliver C, Hawkins A, Dalgleish T. 2010 Can you feel the beat? Interoceptive awareness is an interactive function of anxiety- and depression-specific symptom dimensions. Behav. Res. Ther. 48, 1133–1138. (doi:10.1016/j.brat.2010.07.006) PMC2964892 20692645

[88] Schmidt NB , Trakowski J. 2004 Interoceptive assessment and exposure in panic disorder: a descriptive study. Cognit. Behav. Pract. 11, 81–92. (doi:10.1016/S1077-7229(04)80010-5)

[89] Domschke K , Stevens S, Pfleiderer B, Gerlach AL. 2010 Interoceptive sensitivity in anxiety and anxiety disorders: an overview and integration of neurobiological findings. Clin. Psychol. Rev. 30, 1–11. (doi:10.1016/j.cpr.2009.08.008) 19751958

[90] Boswell JF , Farchione TJ, Sauer-Zavala S, Murray HW, Fortune MR, Barlow DH. 2013 Anxiety sensitivity and interoceptive exposure: a transdiagnostic construct and change strategy. Behav. Ther. 44, 417–431. (doi:10.1016/j.beth.2013.03.006) PMC3727659 23768669

[91] Meyerholz L , Irzinger J, Witthöft M, Gerlach AL, Pohl A. 2019 Contingent biofeedback outperforms other methods to enhance the accuracy of cardiac interoception: a comparison of short interventions. J. Behav. Ther. Exp. Psychiatry 63, 12–20. (doi:10.1016/j.jbtep.2018.12.002) 30557753

[92] Wareing L , Readman MR, Longo MR, Linkenauger SA, Crawford TJ. 2024 The utility of heartrate and heartrate variability biofeedback for the improvement of interoception across behavioural, physiological and neural outcome measures: a systematic review. Brain Sci. 14, 579. (doi:10.3390/brainsci14060579) PMC11487402 38928579

[93] Lehrer P , Kaur K, Sharma A, Shah K, Huseby R, Bhavsar J, Sgobba P, Zhang Y. 2020 Heart rate variability biofeedback improves emotional and physical health and performance: a systematic review and meta-analysis. Appl. Psychophysiol. Biofeedback 45, 109–129. (doi:10.1007/s10484-020-09466-z) 32385728

[94] Herhaus B , Siepmann M, Kahaly GJ, Conrad R, Petrowski K. 2022 Effect of a biofeedback intervention on heart rate variability in individuals with panic disorder: a randomized controlled trial. Psychosom. Med. 84, 199–209. (doi:10.1097/PSY.0000000000001031) 34654028

[95] Gross JJ . 2015 Emotion regulation: current status and future prospects. Psychol. Inq. 26, 1–26. (doi:10.1080/1047840X.2014.940781)

[96] Gross JJ , Jazaieri H. 2014 Emotion, emotion regulation, and psychopathology: an affective science perspective. Clin. Psychol. Sci. 2, 387–401. (doi:10.1177/2167702614536164)

[97] O'Toole MS , Pedersen AD. 2011 A systematic review of neuropsychological performance in social anxiety disorder. Nord. J. Psychiatry 65, 147–161. (doi:10.3109/08039488.2011.565801) 21428862

[98] Banaszkiewicz M . 2017 It is good to be stressed: improving performance and body responses by stress reappraisal. J. Educ. Health Sport 7, 447–453. (doi:10.5281/zenodo.495784)

[99] Brooks AW . 2014 Get excited: reappraising pre-performance anxiety as excitement. J. Exp. Psychol. Gen. 143, 1144–1158. (doi:10.1037/a0035325) 24364682

[100] Jamieson JP , Nock MK, Mendes WB. 2013 Changing the conceptualization of stress in social anxiety disorder: affective and physiological consequences. Clin. Psychol. Sci. 1, 363–374. (doi:10.1177/2167702613482119)

[101] Beltzer ML , Nock MK, Peters BJ, Jamieson JP. 2014 Rethinking butterflies: the affective physiological, and performance effects of reappraising arousal during social evaluation. Emotion 14, 761–768. (doi:10.1037/a0036326) 24749642

[102] Brown KW , Ryan RM. 2003 The benefits of being present: mindfulness and its role in psychological well-being. J. Pers. Soc. Psychol. 84, 822–848. (doi:10.1037/0022-3514.84.4.822) 12703651

[103] Baer RA . 2003 Mindfulness training as a clinical intervention: a conceptual and empirical review. Clin. Psychol. Sci. Pract. 10, 125–143. (doi:10.1093/clipsy.bpg015)

[104] Goyal M *et al.* 2014 Meditation programs for psychological stress and well-being: a systematic review and meta-analysis. JAMA Intern. Med. 174, 357–368. (doi:10.1001/jamainternmed.2013.13018) PMC4142584 24395196

[105] Kim MK , Lee KS, Kim B, Choi TK, Lee S-H. 2016 Impact of mindfulness-based cognitive therapy on intolerance of uncertainty in patients with panic disorder. Psychiatry Investig. 13, 196–202. (doi:10.4306/pi.2016.13.2.196) PMC4823195 27081380

[106] Koszycki D , Thake J, Mavounza C, Daoust J-P, Taljaard M, Bradwejn J. 2016 Preliminary investigation of a mindfulness-based intervention for social anxiety disorder that integrates compassion meditation and mindful exposure. J. Altern. Complement. Med. 22, 363–374. (doi:10.1089/acm.2015.0108) 27070853

[107] de Lima-Araujo GL , de Sousa Jr GM, Mendes T, Demarzo M, Farb N, de Araujo DB, de Sousa MBC. 2022 The impact of a brief mindfulness training on interoception: a randomized controlled trial. PLoS ONE 17, e0273864. (doi:10.1371/journal.pone.0273864) PMC9451078 36070308

[108] Parkin L *et al.* 2014 Exploring the relationship between mindfulness and cardiac perception. Mindfulness 5, 298–313. (doi:10.1007/s12671-012-0181-7)

[109] Mehling WE , Price C, Daubenmier JJ, Acree M, Bartmess E, Stewart A. 2012 The multidimensional assessment of interoceptive awareness (MAIA). PLoS ONE 7, e48230. (doi:10.1371/journal.pone.0048230) PMC3486814 23133619

[110] Daubenmier J , Sze J, Kerr CE, Kemeny ME, Mehling W. 2013 Follow your breath: respiratory interoceptive accuracy in experienced meditators. Psychophysiology 50, 777–789. (doi:10.1111/psyp.12057) PMC3951998 23692525

[111] Nesvold A , Fagerland MW, Davanger S, Ellingsen Ø, Solberg EE, Holen A, Sevre K, Atar D. 2012 Increased heart rate variability during nondirective meditation. Eur. J. Prev. Cardiol. 19, 773–780. (doi:10.1177/1741826711414625) 21693507

[112] Dobkin PL , Irving JA, Amar S. 2012 For whom may participation in a mindfulness-based stress reduction program be contraindicated? Mindfulness 3, 44–50. (doi:10.1007/s12671-011-0079-9)

[113] Quadt L , Garfinkel SN, Mulcahy JS, Larsson DE, Silva M, Jones A-M, Strauss C, Critchley HD. 2021 Interoceptive training to target anxiety in autistic adults (ADIE): a single-center, superiority randomized controlled trial. EClinicalMedicine 39, 101042. (doi:10.1016/j.eclinm.2021.101042) PMC8350004 34401684

